# Genetic Variation of SARS Coronavirus in Beijing Hospital

**DOI:** 10.3201/eid1005.030875

**Published:** 2004-05

**Authors:** Dongping Xu, Zheng Zhang, Fuliang Chu, Yonggang Li, Lei Jin, Lingxia Zhang, George F. Gao, Fu-Sheng Wang

**Affiliations:** * Beijing 302 Hospital, Beijing, China; † University of Oxford, Headington, Oxford, United Kingdom

**Keywords:** SARS coronavirus,genetic variation, quasispecies,spike glycoprotein gene

## Abstract

To characterize genetic variation of severe acute respiratory syndrome–associated coronavirus (SARS-CoV) transmitted in the Beijing area during the epidemic outbreak of 2003, we sequenced 29 full-length S genes of SARS-CoV from 20 hospitalized SARS patients on our unit, the Beijing 302 Hospital. Viral RNA templates for the S-gene amplification were directly extracted from raw clinical samples, including plasma, throat swab, sputum, and stool, during the course of the epidemic in the Beijing area. We used a TA-cloning assay with direct analysis of nested reverse transcription–polymerase chain reaction products in sequence. One hundred thirteen sequence variations with nine recurrent variant sites were identified in analyzed S-gene sequences compared with the BJ01 strain of SARS-CoV. Among them, eight variant sites were, we think, the first documented. Our findings demonstrate the coexistence of S-gene sequences with and without substitutions (referred to BJ01) in samples analyzed from some patients.

A novel severe acute respiratory syndrome–associated coronavirus (SARS-CoV) has been implicated as the causative agent of a worldwide outbreak of SARS during the first 6 months of 2003 (*1*–*3*). From March 4 to June 18, Beijing had 2,521 cases and 191 deaths from SARS (*4*). Because of the poor fidelity of RNA-dependent RNA polymerase, genetic variation typically forms a heterogeneous virus pool in RNA virus populations, including coronaviruses such as mouse hepatitis virus (MHV) (*5*,*6*). This feature makes viruses highly adaptable and contributes to difficulties in preventing and controlling viral disease. SARS-CoV, a single-stranded RNA virus, has been reported with relatively less variability in analyses of a limited number of viral isolate collections (*7*–*10*). Furthermore, no SARS-CoV quasispecies have been documented, as they have been in many other RNA viruses, including hepatitis C virus (HCV) (*11*), HIV (*12*), and MHV (*6*).

During the SARS outbreak in Beijing, 132 SARS patients were hospitalized and treated on our unit at Beijing Hospital, including the first cluster of case-patients in the area (*13*). To characterize genetic variation among SARS-CoV transmitted in the Beijing area, we sequenced 29 full-length S genes of SARS-CoV from 20 hospitalized SARS patients, since S glycoprotein plays a key role in virus-host interaction and is predicted to be the main target of immune response (*14*). Samples that were analyzed represented the timespan of the epidemic. To exclude culture-derived artifacts and estimate mutational heterogeneity, viral RNA was directly extracted from raw clinical samples, and a TA-cloning assay was used with direct analysis of reverse transcriptase–polymerase chain reaction (RT-PCR) products. We compared these sequences with all previously documented S-gene sequences of SARS-CoV.

## Materials and Methods

### Patients and Samples

**Figure 1:**
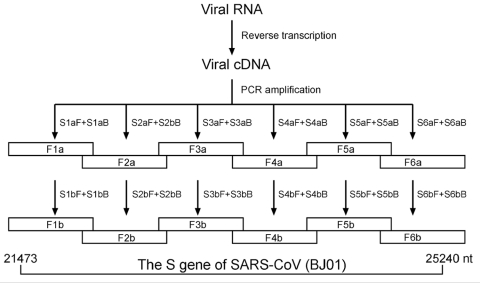
Diagram showing amplification of six overlapping fragments covering full-length spike gene sequence of severe acute respiratory syndrome–associated coronavirus by nested reverse transcriptase–polymerase chain reaction.

All patients in the study were hospitalized on our unit with a confirmed diagnosis of SARS. Samples from patients included plasma, throat swab, sputum, and stool; these were stored at –70°C for extraction of viral RNA. A total of 64 RNA samples from 28 SARS-CoV–positive patients (detected by using BNI primers recommended by the World Health Organization [*15*]) were initially used in S-gene amplification, but only those that generated all six overlapping fragments covering the full-length S-gene sequence (see Nested RT-PCR below and Figure 1) were included in the sequence analysis. As a result, 29 RNA samples from 20 patients were included in the study (Table 1). All patients had received ribavirin and steroid combination therapy.

**Table 1:** Clinical backgrounds of patients and sample collection

Patient no.	Age (y)	Sex^a^	Onset date	Hospitalized date	Specimen no.^b^	Sampling date
1	53	M	2/28	3/05	SW6	3/06
2	32	M	3/08	3/08	SW17	3/09
3	32	F	3/20	4/04	PL1	4/07
4	20	M	3/21	4/06	PL10	4/07
					PL17	4/22
					SP4	5/03
5	33	M	3/28	4/04	PL9	4/07
					SP1	5/03
6	59	M	3/30	4/06	PL5	4/07
					SP9	5/12
7	52	M	3/30	4/04	PL7	4/07
8	59	M	3/30	4/06	PL8	4/07
9	19	F	4/01	4/12	PL15	4/22
					SP32	4/26
10	73	M	4/02	4/03	PL6	4/07
					SP62	4/18
					SW73	4/21
11	45	F	4/04	4/04	SP67	4/18
12	26	M	4/08	4/18	SW76	4/21
13	31	M	4/08	4/14	ST123	4/26
14	32	M	4/09	4/18	PL57	4/21
					SW77	4/22
15	39	M	4/10	4/10	SP61	4/18
16	31	F	4/10	4/12	PL59	4/30
17	46	F	4/20	4/21	SP28	4/26
18	48	M	4/20	4/22	SP43	4/24
19	38	M	4/22	4/26	SP13	5/03
					ST158	4/30
20	27	M	5/10	5/11	SP8	5/12

^a^M, male; F, female. ^b^First two letters indicate source of sample: SW, throat swab; PL, plasma; SP, sputum; ST, stool.

### RNA Extraction

RNA extraction was performed in a biosafety level 3 (P3) laboratory. RNA was extracted directly from plasma samples. Sputum samples were shaken for 30 min with an equal volume of 1.0% acetylcysteine and 0.9% sodium chloride, followed by isolating supernatant by centrifuging (10,000*g* x 3 min). Throat swab and stool samples were suspended with phosphate-buffered saline (PBS) containing 10 U/mL RNasin (Promega, Madison, WI) and shaken for 10 min, followed by isolating supernatant by centrifuging as mentioned above. RNA was extracted according to the manufacturer’s instructions by using the QIAamp Viral RNA Mini Kit (Qiagen, Hilden, Germany).

### Nested RT-PCR

Screening RNA for SARS-CoV was based on the method by Drosten et al. (*1*). For the S-gene amplification, 18 pairs of primers were designed by using MacVactor computer software (Accelrys Inc, San Diego, CA) based on the BJ01 strain of SARS-CoV (GenBank accession no. AY278488) (*16*). Among them, six pairs (sense/antisense: S1aF/S1aB, S2aF/S2aB, S3aF/S3aB, S4aF/S4aB, S5aF/S5aB, S6aF/S6aB) were used as outer primers, six pairs (sense/antisense: S1bF/S1bB, S2bF/S2bB, S3bF/S3bB, S4bF/S4bB, S5bF/S5bB, S6bF/S6bB) were used as inner primers, and six pairs (sense/antisense: S1cF/S1cB, S2cF/S2cB, S3cF/S3cB, S4cF/S4cB, S5cF/S5cB, S6cF/S6cB) were designed for direct RT-PCR product sequencing. The sequences covering the full-length S gene were amplified separately as six overlapping fragments (F1b, F2b, F3b, F4b, F5b, and F6b) (Figure 1). The one-step RT-PCR Kit (Qiagen) was used for reverse transcription and the first round of PCR amplification with outer primers. Thermal cycling consisted of 50°C for 30 min; 95°C for 15 min; 10 cycles of 95°C for 30 s, 57.5°C for 30 s (decreasing by 1.5°C every other cycle), 72°C for 1 min; 40 cycles of 95°C for 30 s, 54°C for 30 s, 72°C for 1 min. Afterwards, 2 μL of the product was used as a template for the second round of PCR amplification in 100-μL volume with inner primers with Taq DNA polymerase (MBI Fermentas, Hanover, MD). Thermal cycling consisted of 30 cycles of 95°C for 25 s, 54°C for 25 s, 72°C for 50 s. In some cases, Transcript III RNase H^–^ Reverse Transcriptase (Invitrogen, Carlsbad, CA) was used for reverse transcription, according to the manufacturer’s instructions. The next two rounds of PCR amplification were performed by using Platinum Pfx DNA Polymerase with a higher fidelity (Invitrogen). The reaction condition was set as above, with a twofold elongation at 68°C instead of 72°C. All reactions were carefully carried out to avoid contamination.

### TA-Cloning

RT-PCR products were purified by QIAquick PCR Purification Kit (Qiagen) or QIAquick Gel Extraction Kit (Qiagen), with a final volume of 30 μL of elution. The ligation and transformation were performed according to the manufacturer’s instructions by using pGEM-T Vector System II (Promega). Transformants were selected in LB-agar plate containing 100 μg of ampicillin, 100 μg of 5-bromo-4-chloro-3-indolyl β-L-fucopyranoside (X-gal), and 200 μg of isopropylthiogalactoside. *Escherichia coli* from white clones was added to 5 mL of LB culture for overnight growing at 37°C with vigorous shaking. Plasmid was purified by QIAprep Spin Miniprep Kit (Qiagen). The recombinant plasmids for sampling sequence analysis were screened by electrophoresis in 1% agarose containing 0.5 μg/mL of ethidium bromide.

### Sequencing and DNA Analysis

For each S-gene fragment, four to six clones were screened. To verify variations, 5–50 additional clones generated from independently prepared, RNA-derived RT-PCR products were sequenced in two to four independent experiments. The cloned plasmids were prepared from different RT-PCR products and were directly sequenced for confirmation. DNA sequences were obtained with the use of an automated ABI 377 sequencer (Applied Biosystems Inc., Foster City, CA). For cloned plasmids, SP6 and T7 primers were used for two-directional sequencing reactions. For PCR products, specific primers (sense: S1cF–S6cF; antisense: S1cB–S6cB) were used for two-directional sequencing reactions. Analysis and comparison of nucleotide and amino acid sequences were carried out with the DNASTAR computer software (DNASTAR Inc., Madison, WI). The S gene sequence of BJ01 strain was taken as the reference for variation analysis.

## Results

With the designed six pairs of primers, all six overlapping S-gene fragments were amplified by nested RT-PCR from 29 RNA samples. However, most RNA samples initially included in the study, though positive for SARS-CoV with BNI primers, failed to simultaneously generate all six overlapping S-gene fragments and were excluded from further sequence analysis. Disintegration of the virus and low viral load in the raw samples likely accounted for these failures.

**Figure 2:**
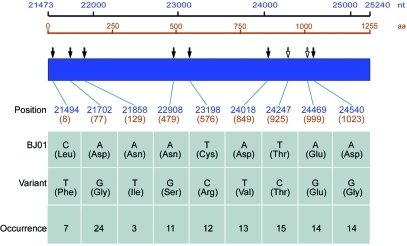
Variants identified from 29 full-length S genes of severe acute respiratory syndrome–associated coronavirus from 20 SARS patients in comparison with BJ01 strain (GenBank accession no. AY278488). The nucleotide positions are numbered according to the sequence of BJ01 strain. Numbers start from the beginning of the genome, but the amino acid numbers start from the S protein. The filled arrows represent nonsynonymous mutations, and the hollow arrows represent synonymous ones. The occurrence indicates the frequency of the variant nucleotide at the given site of the identified 29 entire S genes.

One hundred and thirteen sequence variations distributed in nine variant sites were identified in analyzed sequences that were compared to the reference BJ01 strain of SARS-CoV. BJ01 is an isolate from a tissue-culture propagated sample (*16*) and is used as reference strain in other studies (*9*,*10*). With the exception of one site (position 21702), other variant sites have not, to our knowledge, been documented in humans. Seven of nine variant sites were nonsynonymous. Figure 2 shows the identified variant sites compared to the reference sequence.

## Discussion

We identified novel variant sites and the coexistence of sequences with and without S-gene substitutions in SARS-CoV. Theoretically, a replicating RNA virus expresses a range of genetic and phenotypic variants and has the potential to generate novel virions, which may be selected in response to environmental pressures. RNA viruses generally tolerate high levels of mutagenesis because of their limited genetic complexity (*17*). Mutations have the potential to be pathogenic (e.g., giving the virus immunity to neutralizing antibodies, cytotoxic T cells, or antiviral drugs [*18**–**20*]). The dynamics of error copying and sequence decomposition are time-dependent. In HIV infection, for example, one adaptive substitution in the *env* gene occurred every 3.3 months or 25 viral generations, averaging across patients (*21*).

In our study, a higher variation frequency in the S gene was identified for SARS-CoV compared to previous reports (*7*–*10*). This difference may be due to a broader sample collection covering a longer timespan of infection. In addition, since virus isolates were not passaged in culture, the whole mutant repertoire is more likely to be detected, since no reverse mutation occurs in cell culture. Our observation most likely reflected the real situation in vivo. Variations were unlikely to result from Taq polymerase errors, since we repeated the experiments for all variations from preparing independent RNA and RT-PCR products and used Platinum Pfx DNA polymerase, which has a high fidelity, to confirm the results in some cases. We could not exclude the possibility that some variations were from defective genomes. However, the fact that the variations remained detectable in the sequences from two or three specimens of the same patient, obtained at different times, suggested that these variations might be active and extensible in vivo.

Sequences with and without substitutions (referred to BJ01) were simultaneously detected in the sequences from seven samples, which suggests the existence of SARS-CoV quasispecies. Furthermore, S-gene sequences from different samples collected at different times from the same patient showed similar, but not exactly identical, variation profiles in four participants (patients 4, 5, 6, and 19 in Table 1); this implies that a dynamic mutational process may exist in vivo. Table 2 summarizes the variations occurring in 29 analyzed S-gene sequences from 20 individual SARS patients.

**Table 2:** Variation in S-gene sequences from 20 individual SARS patients^a,b^

Pt. no.	Samp. no.	21494	21702	21858	22908	23198	24018	24247	24469	24540	
		C→T	A→G	A→T	A→G	T→C	A→T	T→C	A→G	A→G	
1	SW6	–	–	–	–	–	–	–	–	–	
2	SW17	9/2^c^	+	–	–	–	–	+	–	–	
3	PL1	8/39	8/43	48/2	+	+	+	+	+	+	
4	PL10	14/7	+	–	+	+	2/8	+	+	+	
	PL17	+	+	–	–	–	+	+	+	+	
	SP4	–	+	–	+	–	+	+	+	+	
5	PL9	+	+	–	+	+	+	+	+	+	
	SP1	–	+	–	+	+	–	+	+	+	
6	PL5	–	+	–	+	+	8/4	+	+	+	
	SP9	–	+	–	+	+	+	+	+	+	
7	PL7	–	+	–	+	+	4/6	+	+	+	
8	PL8	7/28	+	33/2	+	+	+	+	+	+	
9	SP15	–	+	–	–	–	–	–	–	–	
	SP32	–	+	–	–	–	–	–	–	–	
10	SP6	–	–	–	–	–	–	–	–	–	
	SP62	–	–	–	–	–	–	–	–	–	
	SW73	–	–	–	–	–	–	–	–	–	
11	SP67	–	+	–	–	–	–	–	–	–	
12	SW76	–	+	–	–	–	–	–	–	–	
13	ST123	–	+	–	–	–	–	–	–	–	
14	PL57	–	+	–	–	+	+	+	+	+	
	SW77	–	+	–	–	+	+	+	+	+	
15	SP61	–	+	–	–	–	–	–	–	–	
16	PL59	–	–	–	–	–	–	–	–	–	
17	SP28	–	+	–	–	–	–	–	–	–	
18	SP43	–	+	–	–	–	–	–	–	–	
19	ST158	–	+	–	+	+	+	+	+	+	
	SP13	19/4	14/10	10/13	+	+	+	6/16	+	14/8	
20	SP8	–	+	–	–	–	–	–	–	–	

^a^The results were determined by analysis of cloned sequences; + represents that nucleotide substitution at the variant site is detected and – represents that the nucleotide at the site is identical to the one of BJ01 reference sequence in all analyzed sequences. ^b^SARS, severe acute respiratory syndrome; SW, throat swab; PL, plasma; SP, sputum; ST, stool. ^c^The numbers represent the ratio of reference to variant nucleotide detected at the site from the analyzed cloned sequences.

One nonsynonymous change observed at position A1023G is within the heptad repeat (HR) domains, which is thought to be important for virus entry, and previous study on MHV showed that it would have some effect on virus infection (*22*). At this stage, we cannot rule out the possibility that this change affects the biological outcome of the virus, but further experiments need to be addressed in the near future.

We observed the coexistence of the S-gene sequences with and without substitutions and time-dependent variation profile in some patients. These observations suggest the possible existence of SARS-CoV quasispecies in an acute infection. In this study, however, the limitation of clinical sample collection and difficulty in directly amplifying full-length S gene from raw clinical samples restricted further extensive study for dynamic mutant distributions of the virus. In addition, the sequencing clone number was conditioned by the scale of the project, and this may have led to some minor variant sequences escaping analysis. Another factor possibly affecting the stability of the viral genome is the administration of the antiviral drug ribavirin. That ribavirin enhances mutagensis of RNA viruses has been addressed (*23*). Therefore, the artificial effect of ribavirin on the SARS-CoV mutant spectrum remains to be clarified.

The genetic variation of SARS-CoV remains limited in relation to many other RNA viruses such as HIV-1, HCV, and MHV. The probable reason is that SARS-CoV only causes an acute, self-limited infection, which may prevent persistent long-term mutant development in vivo as occurs in chronic RNA viral infections. Notably, some modules in the S protein remain conserved, e.g., the fusion-important HR domains. Although some variations may predict changes of protein functional features, no obvious correlation exists between mutation and clinical disease manifestation from the limited data reported here. Instead, the variation profile was closely correlated with epidemiography; e.g., patients 3–8 were infected in one hospital.

In conclusion, we report here some new variant sites in the S gene of coronavirus and possible existence of SARS-CoV quasispecies in some patients, though in limited numbers. This knowledge furthers our understanding of this emerging virus.
